# Assessing Productivity Development of Public Hospitals: A Case Study of Shanghai, China

**DOI:** 10.3390/ijerph17186763

**Published:** 2020-09-16

**Authors:** Juan Du, Shuhong Cui, Hong Gao

**Affiliations:** School of Economics and Management, Tongji University, 1239 Siping Road, Shanghai 200092, China; dujuan@tongji.edu.cn (J.D.); cuishuhong@tongji.edu.cn (S.C.)

**Keywords:** health care, Malmquist–Luenberger (M-L) productivity index, public hospitals, total factor productivity (TFP)

## Abstract

As the main provider of medical services for the general public, the productivity changes of public hospitals directly reflect the development of the healthcare system and the implementation effect of medical reform policies. Using the dataset of 126 public hospitals in China from 2013 to 2018, this paper improves the existing literature in both index selection and model formulation, and examines public hospitals’ total factor productivity (TFP) growth. Empirical results not only demonstrate the trend of productivity development but also point out the directions in how to improve the current running status. Our study demonstrates that there were no obvious productivity fluctuations in public hospitals during the recent observing years, indicating that the performance of China’s public health system was generally acceptable in coping with fast-growing medical demand. However, the effect of public hospital reform has not been remarkably shown; thus, no significant productivity improvement was observed in most hospitals. Tertiary hospitals witnessed a slight declining trend in TFP, while secondary hospitals showed signs of rising TFP. To effectively enhance the overall performance of public hospitals in China, practical suggestions are proposed from the government and hospital levels to further promote the graded medical treatment system.

## 1. Introduction

In order to cope with the growing demand of healthcare services and to meet health-related goals, it is important to develop a scientific and effective assessment of health system performance as well as to investigate the efficient use of limited medical resources. Compared to developed countries, China is facing a more serious shortage of health resources [1]. This situation is possibly caused by two reasons: First, the number of medical staff makes it difficult to meet the increasing healthcare needs [2]; second, medical resources have not been reasonably allocated and used [3]. Associated with social economic transitions such as aging and urbanization, the general public’s basic health needs have experienced a rapid growth and presented diversified characteristics in China [4,5]. These situations have posed great challenges to establishing and perfecting the medical service system.

To deal with these challenges, China has promoted health care reform to modify the current structure with the goal to reduce the burden of residents’ medical expenses and to enhance productivity, quality, and accessibility of the health system [6,7]. A key content of China’s health care reform is building and popularizing the graded medical treatment system, which is supposed to be a crucial measure to realize equitable allocation of healthcare resources and to ensure equal access to basic medical services [8,9].

As the main body of China’s medical service system, public hospitals undertake most of the medical treatment and play a leading role in scientific research, teaching, emergency response, and other social services [10,11]. Due to the importance of public hospitals, great attention has been paid to evaluating their capability of utilizing medical resources into healthcare services. Therefore, in order to examine the effect of medical reform, it is necessary to develop a scientific method to assess the longitudinal performance change of different tiers of public hospitals.

The main ways to measure productivity change include indicator methods and programming models. Indicator methods use representative indexes to assess hospitals’ performance, such as the ratio of outputs to inputs [12,13,14] and real costs per case-mixed-adjusted discharge [15]. Although easy to operate, indicator methods have limitations in integrating multiple inputs and multiple outputs into a single index. First, the weights used to combine various metrics are user-determined and can be subjective. Second, not all metrics are suitable to be converted into financial measures, making it difficult to aggregate them into one indicator.

To tackle the limitations existing in indicator methods, some studies propose programming models for productivity change measurement [16]. Under the framework of data envelopment analysis (DEA) [17], Cheng et al. [18] apply DEA and the Malmquist index to examine productivity changes from 2010 to 2012 of 114 sample county hospitals located in Henan province, China. van Ineveld et al. [19] study how hospital DEA efficiency has developed since the start of the Dutch health system reform in 2005 to 2010. These two studies only consider input reductions, but ignore the potential in output-side improvement.

Follow-up studies generate productivity indicators that include both input contractions and output expansions. Barros et al. [20] employ the directional distance function (DDF) to calculate the Luenberger productivity index of sample Portuguese hospitals. The main advantage of DDF is simultaneously determining the input decreases and output increases in a given direction of an observed hospital to reach the production frontier. Zhang et al. [21] construct a non-oriented slacks-based DEA measure to estimate dynamic change in efficiency of medium-sized public hospitals in Japan. Estimated through both input- and output-oriented measures, Liu et al. [22] also combine a slacks-based super-efficiency DEA model with the Malmquist productivity index to evaluate the health expenditure efficiency in rural China from 2007 to 2016. Although the above literature have improved the method to some extent, the evaluation criteria are not comprehensive enough. The metrics chosen in these previous studies only reflect the dimension of hospitals’ operation scale such as personnel, beds, and cases, but do not cover the medical service status, which is another significant dimension of hospital performance.

To deal with the above-mentioned limitations, this paper tries to improve the existing research in both index selection and model formulation. Besides the metrics concerning operating scale, we also take into account measures that reflect the status quo of medical services, including the average length of stay and the daily average cost per discharge, which are regarded as undesirable output measures. With respect to methodology, this study modifies the conventional DDF model to calculate the Malmquist–Luenberger (M-L) index, which is used to measure the total factor productivity (TFP) change. Empirical results from a dataset consisting of 126 public hospitals located in the metropolis of Shanghai illustrate that public hospitals have experienced a steady productivity development from 2013 to 2018. It is indicated that public hospitals are doing not so badly in dealing with fast-growing medical needs, but the effect of China’s health care reform on public hospitals has not been fully demonstrated. Different tiers of public hospitals have shown differences in productivity change. Tertiary hospitals witnessed a slightly declining trend in TFP, while secondary hospitals showed signs of rising. These observations imply that the graded medical system needs to be further popularized in China.

The remainder of this paper is organized as follows: Section 2 presents the methodology, followed by presentation of data and results in Section 3. Section 4 discusses on the empirical results. Section 5 concludes.

## 2. Methods

The concept of Malmquist productivity index (MPI) was first introduced by Malmquist [23] as a measurement of productivity change over time. Caves et al. [24] begin to adopt distance functions in characterizing this index, and use the geometric mean of two-adjacent-period indexes to measure productivity growth. In this study, besides the metrics reflecting a hospital’s operating scale such as personnel and beds, we also take medical service conditions into consideration. Specifically, the average length of stay and the daily average cost per discharge are selected to represent this dimension, which are supposedly the smaller the better. Thus these two metrics are regarded as undesirable output measures.

In the context of health care, suppose that there are a set of *n* hospitals, and each of them is denoted by $DMU_{j}$ (*j* = 1, ..., *n*). At time period *t*, hospital *j* utilizes *m* inputs to produce *s* desirable outputs and *q* undesirable outputs, where its *i*th input, *r*th desirable output, and *p*th undesirable output are denoted by $x_{ij}^{t}$ (*i* = 1, ..., *m*), $y_{rj}^{t}$ (*r* = 1, ..., *s*) and $b_{pj}^{t}$ (*p* = 1, ..., *q*), respectively. In order to evaluate the productivity growth in the presence of undesirable outputs, Chung et al. [16] use the output-oriented directional distance function (DDF), defined by Chambers et al. [25], to characterize the distance function of MPI and then develop the Malmquist–Luenberger (M-L) index. 

To assess hospitals’ performance in a more comprehensive way, this study modifies the conventional DDF model proposed by Chung et al. [16], and evaluates from both ends of inputs and outputs. Using $DMU_{o}$ from period $t_{2}$ as an example, our modified DDF model (1) seeks for the largest feasible increase in desirable outputs compatible with the greatest feasible reduction in inputs and undesirable outputs. In Model (1), $D_{o}^{t_{1}}\left(x_{o}^{t_{2}},y_{o}^{t_{2}},b_{o}^{t_{2}};-x_{o}^{t_{2}},y_{o}^{t_{2}},-b_{o}^{t_{2}}\right)$ represents the greatest compatible change of data from period $t_{2}$ with respect to the productivity technology of period $t_{1}$.

(1) $$\begin{array}{ll} D_{o}^{t_{1}} & \left(x_{o}^{t_{2}},y_{o}^{t_{2}},b_{o}^{t_{2}};-x_{o}^{t_{2}},y_{o}^{t_{2}},-b_{o}^{t_{2}}\right)=max\beta_{o}\\ s.t. & \overset{n}{\underset{j=1}{\sum }}\lambda_{j}^{t_{1}}x_{ij}^{t_{1}}\leq \left(1-\beta_{o}\right)x_{io}^{t_{2}},\;i=1,\ldots ,m\\ & \overset{n}{\underset{j=1}{\sum }}\lambda_{j}^{t_{1}}y_{rj}^{t_{1}}\geq \left(1+\beta_{o}\right)y_{ro}^{t_{2}},\;r=1,\ldots ,s\\ & \overset{n}{\underset{j=1}{\sum }}\lambda_{j}^{t_{1}}b_{pj}^{t_{1}}\leq \left(1-\beta_{o}\right)b_{po}^{t_{2}},\;p=1,\ldots ,q\\ & \lambda_{j}^{t_{1}}\geq 0,\;j=1,\ldots ,n \end{array}$$

Let $t_{1}=t_{2}=t$ or t+1 in Model (1), and we get two single-period measures as $D^{t}\left(x^{t},y^{t},b^{t};-x^{t},y^{t},-b^{t}\right)$ and $D^{t+1}\left(x^{t+1},y^{t+1},b^{t+1};-x^{t+1},y^{t+1},-b^{t+1}\right)$. If we let $t_{1}=t$, $t_{2}=t+1$, or $t_{1}=t+1$, $t_{2}=t$, two adjacent-period measures are obtained as $D^{t}\left(x^{t+1},y^{t+1},b^{t+1};-x^{t+1},y^{t+1},-b^{t+1}\right)$ and $D^{t+1}\left(x^{t},y^{t},b^{t};-x^{t},y^{t},-b^{t}\right)$. Based on the above measures, the M-L index characterized by the technology of period $t_{1}$ is calculated as follows, where $t_{1}$ = *t* or *t* + 1.

(2) $$ML^{t_{1}}=\frac{1+D^{t_{1}}\left(x^{t},y^{t},b^{t};-x^{t},y^{t},-b^{t}\right)}{1+D^{t_{1}}\left(x^{t+1},y^{t+1},b^{t+1};-x^{t+1},y^{t+1},-b^{t+1}\right)}$$

In a similar way to the MPI construction [24], our M-L index $ML^{t,t+1}$, which measures TFP change, is also defined as the geometric mean of two adjacent-period indexes which are calculated from Formula (2). An M-L index greater than one indicates productivity enhancement. In contrast, an M-L index less than one implies productivity regression. An M-L index that equals one represents unchanged productivity.

(3) $$\begin{array}{l} ML^{t,t+1}=[\frac{1+D^{t}\left(x^{t},y^{t},b^{t};-x^{t},y^{t},-b^{t}\right)}{1+D^{t}\left(x^{t+1},y^{t+1},b^{t+1};-x^{t+1},y^{t+1},-b^{t+1}\right)}\\ {\;\;\;\;\cdot \frac{1+D^{t+1}\left(x^{t},y^{t},b^{t};-x^{t},y^{t},-b^{t}\right)}{1+D^{t+1}\left(x^{t+1},y^{t+1},b^{t+1};-x^{t+1},y^{t+1},-b^{t+1}\right)}]}^{\frac{1}{2}} \end{array}$$

Similar to Färe et al. [26], the M-L index (3) proposed in this paper can be decomposed into the product of two components as shown in Formula (4), measuring efficiency change (EC) and technical change (TC), respectively.

(4) $$\begin{array}{l} ML^{t,t+1}=\frac{1+D^{t}\left(x^{t},y^{t},b^{t};-x^{t},y^{t},-b^{t}\right)}{1+D^{t+1}\left(x^{t+1},y^{t+1},b^{t+1};-x^{t+1},y^{t+1},-b^{t+1}\right)}\\ \;\;\;\cdot [\frac{1+D^{t+1}\left(x^{t+1},y^{t+1},b^{t+1};-x^{t+1},y^{t+1},-b^{t+1}\right)}{1+D^{t}\left(x^{t+1},y^{t+1},b^{t+1};-x^{t+1},y^{t+1},-b^{t+1}\right)}{\cdot \frac{1+D^{t+1}\left(x^{t},y^{t},b^{t};-x^{t},y^{t},-b^{t}\right)}{1+D^{t}\left(x^{t},y^{t},b^{t};-x^{t},y^{t},-b^{t}\right)}]}^{\frac{1}{2}} \end{array}$$

In Formula (4), the first component outside the bracket, which is

(5) $$EC=\frac{1+D^{t}\left(x^{t},y^{t},b^{t};-x^{t},y^{t},-b^{t}\right)}{1+D^{t+1}\left(x^{t+1},y^{t+1},b^{t+1};-x^{t+1},y^{t+1},-b^{t+1}\right)}$$

measures the change of distance between the observation and the technology frontier. This item is referred to as efficiency change (*EC*) of the observed hospital, which determines whether a hospital moves closer or farther from the efficiency frontier over time. Therefore, EC>1 indicates improved efficiency, EC=1 indicates constant efficiency, while EC<1 indicates declined efficiency.

The second component of M-L index characterized in Formula (4).

(6) $$TC=[\frac{1+D^{t+1}\left(x^{t+1},y^{t+1},b^{t+1};-x^{t+1},y^{t+1},-b^{t+1}\right)}{1+D^{t}\left(x^{t+1},y^{t+1},b^{t+1};-x^{t+1},y^{t+1},-b^{t+1}\right)}{\cdot \frac{1+D^{t+1}\left(x^{t},y^{t},b^{t};-x^{t},y^{t},-b^{t}\right)}{1+D^{t}\left(x^{t},y^{t},b^{t};-x^{t},y^{t},-b^{t}\right)}]}^{\frac{1}{2}}$$

represents technical change (*TC*) that measures the frontier shift of production technology from two adjacent periods. It demonstrates whether the overall technology constructed by all observed hospitals is improving or declining across periods. TC>1 implies technological progress, TC=1 implies unchanged technology, and TC<1 implies technological regress.

## 3. Results

### 3.1. Data

The data used in this study consist of 126 public hospitals located in Shanghai from 2013 to 2018, and were collected from the Information Center in Shanghai Municipal Health Commission. Sample hospitals were divided into two categories, namely tertiary hospitals and secondary hospitals. The 126 sample hospitals cover most of the public hospitals in Shanghai, among which 41 or 32.5% are tertiary hospitals and the other 85 or 67.5% are secondary hospitals. 

The evaluation index system is composed of three inputs, three desirable outputs, and two undesirable outputs. Input metrics include the number of medical personnel (X_1_), the actual number of beds (X_2_), and total expenditure (X_3_); desirable output metrics include total revenue (Y_1_), the number of outpatients and emergency patients (Y_2_), and the number of discharged patients (Y_3_); undesirable metrics include the average length of stay (B_1_) and the daily average cost per discharge (B_2_). Here medical personnel account for all registered doctors and nurses, as well as other medical technicians.

### 3.2. Input and Output Growths

**Figure 1:**
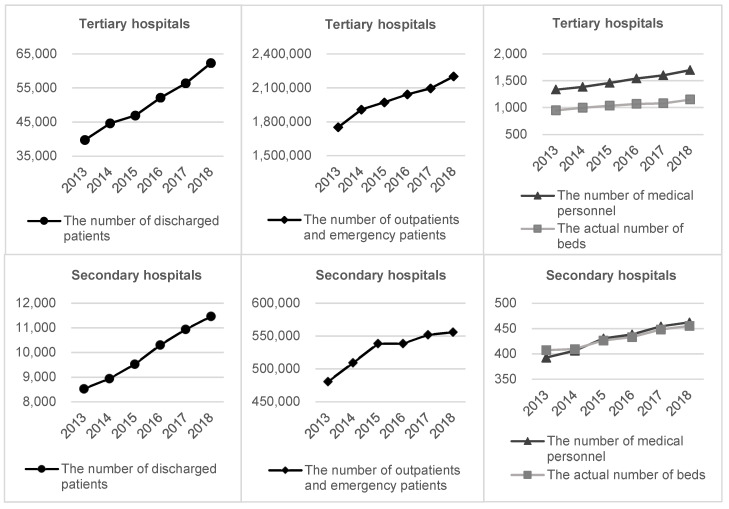
The average operation scales of tertiary and secondary hospitals (2013–2018).

Figure 1 shows that the average scales of both tertiary and secondary hospitals have witnessed an obvious expansion over the observing period from 2013 to 2018. This expansion is most likely due to fast-growing medical needs. In fact, the investment of medical resources and training of medical personnel do not match well with the expansion speed of most hospitals. Figure 1 also indicates that tertiary hospitals have experienced a more rapid growth in size than secondary hospitals. On the input end, the average annual increases of the number of medical personnel and the actual number of beds in tertiary hospitals were 4.93% and 3.92%, while in secondary hospitals the rates were 3.32% and 2.25%. On the output end, the average annual growths of the number of discharged patients and the number of outpatients and emergency patients in tertiary hospitals were 9.43% and 4.68%, while in secondary hospitals were 6.09% and 2.95%. These observations imply that tertiary hospitals undertook some medical services that should otherwise be provided by secondary hospitals.

### 3.3. Productivity Growth

**Figure 2:**
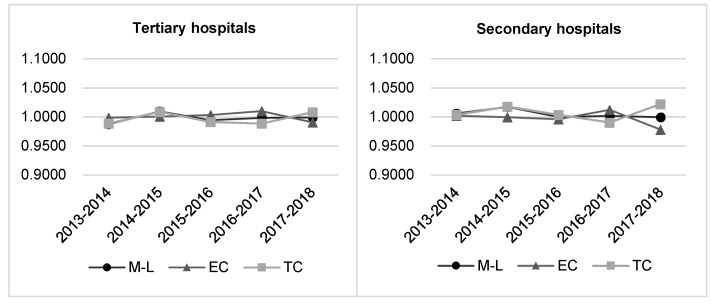
The annual growths of TFP (total factor productivity), EC (efficiency change) and TC (technical change) in tertiary and secondary hospitals.

Table 1 reports the average TFP change characterized by our modified M-L index, as well as the decomposition. From Table 1, it is noticed that in terms of all public hospitals, the average annual growth rates of productivity, EC and TC were 0.25%, −0.15%, and 0.41%, respectively. It indicates that productivity increase of public hospitals was mainly driven by technology progress. By comparing the productivity results of different tiers of hospitals, we find that tertiary hospitals witnessed an average annual decline of 0.23%, while the secondary hospitals experienced a 0.48% increase annually. Figure 2 illustrates that for both tiers of public hospitals, there are no obvious fluctuations in TFP, EC, and TC during the sample years.

In terms of individual efficiency change (EC), tertiary hospitals witnessed an average annual rise of 0.06%, while this rate for secondary hospitals decreased by 0.25%. However, the change of frontier technology which is measured by TC, has shown the opposite trend to EC. Tertiary hospitals suffered an average annual decrease of 0.29%, while secondary hospitals enjoyed a 0.74% progress yearly.

## 4. Discussion

### 4.1. Implications

As aforementioned, both tertiary and secondary hospitals experienced a significant expanding speed during the observation period. This is highly possible to be caused by the fast-growing healthcare demands, which has already put great pressure on public hospitals in providing high-quality medical services.

In China, boundaries between medical institutions are not clear enough. Consequently, functional overlap is common among different tiers of public hospitals. Tertiary hospitals cover all kinds of clinical treatment, from acute severe diseases to common illnesses. It is extremely unbalanced with respect to the volume of medical services between tertiary and other public hospitals, which may result in wasting great amount of healthcare resources and negatively affecting hospitals’ operational efficiency.

The Chinese government has been vigorously promoting graded medical treatment reform since 2015, but with little success. According to the analysis from this study, there are two primary reasons to this phenomenon. First, the general public lacks trust in the medical level of hospitals other than tertiary hospitals. Second, there is no difference in medical charge standard between tertiary hospitals and other level hospitals. Therefore, patients prefer tertiary hospitals where they believe can receive better clinical treatment without paying more. Under this circumstance, non-critical patients occupy too much medical resources which are supposed to be allocated to acute severe patients.

From the perspective of TFP change, public hospitals in China did not fluctuate significantly during the sample years with an M-L index around one. This indicates that the overall performance of public hospitals was relatively stable. However, different tiers of hospitals have shown different development trends. Tertiary hospitals began to show signs of declining productivity, while secondary hospitals demonstrated a trend of steady progress. Due to an excessively rapid growth in healthcare demand, it is difficult for tertiary hospitals to meet this high demand with limited medical resources. This mismatch between fast-growing demand and limited resource allocation directly leads to actual operational efficiency of tertiary hospitals being below the expectation. On the other hand, thanks to the policy support and investment in software and hardware from both the state and local governments, the technical gap between secondary and tertiary hospitals is gradually narrowing. Moreover, through strengthening the systematic vocational training of medical staff, the capability and quality of medical services provided by secondary hospitals have been constantly enhanced.

### 4.2. Suggestions

In order to effectively alleviate the demand pressure of tertiary hospitals and promote the graded medical treatment system, the following suggestions are proposed from the government and hospital levels. First of all, public hospitals, especially secondary hospitals, should continue to enhance their medical technology capabilities to eliminate the biggest concern of the vast majority of patients. The basic premise for promoting graded medical treatment is that patients can build trust in the medical technology level of non-tertiary hospitals. In order to achieve this goal, governments at all levels should give more preferential policies to secondary hospitals, including higher financial budget, greater resources investment and more vocational training opportunities. Through these ways, the ability of secondary hospitals to deal with the acute and severe diseases will be remarkably improved. In the long run, the public will gradually reduce the current strong reliance on tertiary hospitals and establish the habit of turning to the nearest secondary hospitals for initial diagnosis.

Second, the public health administration should further motivate the flow of medical talents and medical service cooperation between tertiary hospitals and other hospitals. For example, doctors from tertiary hospitals can provide outpatient services in secondary hospitals at a proper frequency. With the help of high-tech means such as 5G network communication technology, remote joint consultation and real-time operation guidance should be further promoted among various tiers of hospitals. These measures would transfer part of the medical needs of the tertiary hospitals to the secondary hospitals, and effectively alleviate the rapidly increasing burden of diagnosis and treatment of the tertiary hospitals.

In addition to the above-mentioned long-term measures to balance the development of public hospitals at all tiers, one of the feasible plans to instantly push forward graded medical treatment system is to raise the threshold of outpatient and emergency charges in tertiary hospitals. Due to the sensitivity to medical costs, patients are more willing to choose the secondary hospitals for treatment. Meanwhile, the government should encourage graded medical treatment by giving higher proportion of medical insurance reimbursement to patients who have been transferred to the tertiary hospitals through the secondary hospitals. However, it is worth noting that in order to avoid social contradictions, extra caution should be taken in raising the clinical charge standard of tertiary hospitals. Before making a formal decision, public opinions should be widely and fully solicited, and advantages and disadvantages should be carefully weighed.

## 5. Conclusions

Aiming at tackling the limitations in the existing literature, this paper makes improvements in both index selection and model formulation. Besides scale-related metrics, this study also includes indicators that reflect the status quo of medical services. In terms of methodology, a conventional DDF model is adjusted to generate a modified M-L index to measure the TFP change.

Empirical results from 126 public hospitals illustrate that the performance of China’s public health system was generally acceptable in coping with rapidly growing medical needs. However, no significant improvement in productivity has been observed in most public hospitals, indicating that the effect of the public hospital reform has not been notably shown. Moreover, tertiary hospitals witnessed a slightly declining trend in TFP, while secondary hospitals showed signs of rising. These observations demonstrate that in order to enhance the overall performance of public hospitals, the graded medical treatment system needs to be vigorously promoted and popularized in China.

Future research can be extended in the following directions. First, to get a more panoramic view and assessment of the public health system, the dataset should be expanded to more regions and to include community health centers and grass roots hospitals in rural areas. Second, service quality indicators such as mortality and infection rate can be taken into account as a new dimension to reflect hospitals’ comprehensive performance. But these indicators need to be used with great care. Because the possibilities of different tiers of hospitals are quite different in contacting difficult and miscellaneous diseases, the higher the level of medical technology, the more likely the hospitals are to receive critical patients, resulting in the increase of quality-related measures such as mortality and infection rate. One feasible solution in the follow-up study could be to use the degree of difficulty and danger of the diseases from a hospital to adjust its medical quality indicators. A suitable choice for this numerical adjustment is the case mix index (CMI) generated based on diagnosis related groups (DRGs). CMI refers to the average weight of discharged patients in a hospital, which is related to the types of cases admitted to the hospital. The higher the CMI, the greater the diseases’ difficulty and severity.

## Figures and Tables

**Table 1:** The average productivity change and the decomposition.

	All Hospitals			Tertiary Hospitals			Secondary Hospitals		
Year	M-L	EC	TC	M-L	EC	TC	M-L	EC	TC
2013–2014	0.9999	1.0011	0.9988	0.9875	0.9988	0.9887	1.0060	1.0023	1.0037
2014–2015	1.0146	0.9997	1.0148	1.0094	1.0006	1.0088	1.0172	0.9992	1.0178
2015–2016	0.9980	0.9983	0.9996	0.9945	1.0030	0.9915	0.9996	0.9961	1.0035
2016–2017	1.0008	1.0115	0.9897	0.9984	1.0100	0.9886	1.0019	1.0122	0.9903
2017–2018	0.9992	0.9822	1.0177	0.9988	0.9906	1.0084	0.9994	0.9781	1.0221
Geo-mean	1.0025	0.9985	1.0041	0.9977	1.0006	0.9971	1.0048	0.9975	1.0074
